# Ti_3_C_2_‐MXene Partially Derived Hierarchical 1D/2D TiO_2_/Ti_3_C_2_ Heterostructure Electrode for High‐Performance Capacitive Deionization

**DOI:** 10.1002/advs.202204041

**Published:** 2022-11-28

**Authors:** Ningning Liu, Lanlan Yu, Baojun Liu, Fei Yu, Liqing Li, Yi Xiao, Jinhu Yang, Jie Ma

**Affiliations:** ^1^ Research Center for Environmental Functional Materials State Key Laboratory of Pollution Control and Resource Reuse College of Environmental Science and Engineering Tongji University 1239 Siping Road Shanghai 200092 P. R. China; ^2^ College of Resource and Environmental Engineering Guizhou University Guiyang 550025 China; ^3^ College of Marine Ecology and Environment Shanghai Ocean University Shanghai 201306 P. R. China; ^4^ Faculty of Materials Metallurgy and Chemistry Jiangxi University of Science and Technology Ganzhou 341000 P. R. China; ^5^ Institute of Materials Science TU Darmstadt 64287 Darmstadt Germany; ^6^ School of Chemical Science and Engineering Tongji University 1239 Siping Road Shanghai 200092 P. R. China

**Keywords:** capacitive deionization, heterostructure, MXene, partial derivative, TiO_2_ nanowire

## Abstract

Constructing faradaic electrode with superior desalination performance is important for expanding the applications of capacitive deionization (CDI). Herein, a simple one‐step alkalized treatment for in situ synthesis of 1D TiO_2_ nanowires on the surface of 2D Ti_3_C_2_ nanosheets, forming a Ti_3_C_2_‐MXene partially derived hierarchical 1D/2D TiO_2_/Ti_3_C_2_ heterostructure as the cathode electrode is reported. Cross‐linked TiO_2_ nanowires on the surface help avoid layer stacking while acting as the protective layer against contact of internal Ti_3_C_2_ with dissolved oxygen in water. The inner Ti_3_C_2_ MXene nanosheets cross over the TiO_2_ nanowires can provide abundant active adsorption sites and short ion/electron diffusion pathways. . Density functional theory calculations demonstrated that Ti_3_C_2_ can consecutively inject electrons into TiO_2_, indicating the high electrochemical activity of the TiO_2_/Ti_3_C_2_. Benefiting from the 1D/2D hierarchical structure and synergistic effect of TiO_2_ and Ti_3_C_2_, TiO_2_/Ti_3_C_2_ heterostructure presents a favorable hybrid CDI performance, with a superior desalination capacity (75.62 mg g^−1^), fast salt adsorption rate (1.3 mg g^−1^ min^−1^), and satisfactory cycling stability, which is better than that of most published MXene‐based electrodes. This study provides a feasible partial derivative strategy for construction of a hierarchical 1D/2D heterostructure to overcome the restrictions of 2D MXene nanosheets in CDI.

## Graphical abstract

Cross‐linked 1D TiO_2_ nanowires on the surface of 2D Ti_3_C_2_ MXene nanosheets, which contribute to avoiding layer stacking, simultaneously act as the protective layer to prevent access to the dissolved oxygen in water and hinder the oxidation of Ti_3_C_2_ in the interior. TiO_2_/Ti_3_C_2_ heterostructure presents a favorable hybrid capacitive deionization performance, with a superior desalination capacity, fast salt adsorption rate, and satisfactory cycling stability.

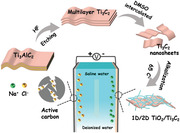

## 1 Introduction

Capacitive deionization (CDI) technology, which separates ions by creating electrical double layers (EDLs) or Faradaic processes, has attracted much attention because of its high work efficiency, reduced energy cost, and easy operation.^[^ ^1^ ^]^ Electrode materials play an essential role in deionization performance. Carbonaceous materials, including activated carbon (AC), carbon nanofibers, and graphene, have broad applications as CDI electrode materials owing to their low cost, large specific surface area (SSA), and superior conductivity.^[^ ^2^ ^]^ However, carbon materials suffer from relatively inferior desalination capacities (usually <25 mg g^−1^) and poor electrochemical stabilities owing to the restriction of the EDLs desalination principle, which thwarts their applications in CDI.^[^ ^3^ ^]^ Alternatively, Faradaic materials, including manganese oxide (MnO_2_),^[^ ^4^ ^]^ Prussian blue and its analogs,^[^ ^5^ ^]^ cobalt oxide (Co_3_O_4_),^[^ ^6^ ^]^ 2D transition metal carbides and nitride (MXene),^[^ ^7^ ^]^ have been widely studied as CDI electrodes recently because of their pseudocapacitive properties. These Faradaic electrodes capture ions through ion intercalation or reversible redox reactions, which are unconstrained by SSA.^[^ ^8^, ^9^ ^]^ Among various Faradaic materials, MXene, which is usually expressed by the formula M*_n_*_+1_X*_n_* T*_x_*, where M is a transition metal (Ti, Mo, etc.), X is carbon and/or nitrogen, and T*_x_* represents the terminating groups (OH, O, and F), exhibits extraordinary potential for CDI applications owing to its combined performance of a high theoretical capacity, remarkable hydrophilic properties, excellent electrical conductivities, large surface area, and tunable surface functional groups.^[^ ^10^, ^11^ ^]^ Srimuk et al. first applied Ti_3_C_2_T*_x_* MXene as an electrode material in a symmetric CDI cell; the average desalination capacity was 13 mg g^−1^ at 1.2 V and sustained the stable performance in 30 CDI cycles.^[^ ^8^ ^]^

There are two factors leading to the restrictions on Ti_3_C_2_T*_x_* MXene desalination performance. First, the restacking of adjacent MXene sheets driven by van der Waals forces reduces the exposure of electrochemical active sites, resulting in a decline in the desalination capacity.^[^ ^12^ ^]^ The construction of 1D/2D hierarchical nanostructures is an efficient method for solving the above agglomeration issue.^[^ ^13^ ^]^ 1D nanomaterials prevent 2D MXene nanosheets from restacking and provide abundant active sites. In particular, the 1D channel with a high length‐to‐diameter ratio will provide rapid charge transfer paths and weak charge transmission resistance during the CDI process.^[^ ^14^ ^]^ A sandwich‐like Ti_3_C_2_T*_x_* MXene/carbon nanotube (CNT) synthetic self‐supporting electrode was synthesized for supercapacitor electrodes by alternately filtrating MXene and CNT dispersions.^[^ ^13b^ ^]^ It had a distensible interlayer space between the MXene sheets. Nevertheless, the layer‐by‐layer assembly strategy tends to have a complicated manufacturing operation and induce weak interactions between 1D/2D nanomaterials.^[^ ^15^ ^]^ The oxidative degradation of MXene is another limitation of its application in CDI. The edges and surface of 2D Ti_3_C_2_T*_x_* MXene are covered by Ti (II) or Ti (III) suboxide or hydroxide/fluoride. They are prone to be oxidized as Ti (IV) oxide (TiO_2_) under ambient air or water,^[^ ^16^ ^]^ which will result in the loss of primordial superior electrical conductivity and destroy the 2D lamellar structure of MXene, resulting in ungratified electrochemical performance.^[^ ^17^ ^]^ Numerous studies have been dedicated to mitigating MXene oxidation by limiting its exposure to water and oxygen.^[^ ^18^ ^]^ Zhao et al. demonstrated that annealing Ti_3_C_2_T*_x_* MXene films at 600 °C under argon gas partially oxidized the surface of Ti_3_C_2_T*_x_* MXene and generated TiO_2_ cladding on the exterior, which prevented further oxidation of the inner layers.^[^ ^19^ ^]^ The treated MXene films exhibited almost no changes in chemical composition and structure even after 10 months of storage in water. Surface partially derived MXene with a relatively light oxidation degree can form derivatives such as transition metal oxides (TMO), on the surface while maintaining a part of MXene inside. The formation of a protective TMO layer passivates the edges and surface of MXene, which can preclude the oxidative degradation of the interior structure and enhance the stability of MXene and is beneficial for extending the layer space and improving the capacitance of MXene.^[^ ^20^ ^]^ Simultaneously, the inherent low electronic conductivity of MXene‐derived TMO is effectively alleviated by residual MXene. The comprehensive properties of the hybrids are dramatically enhanced via the cooperative effect of MXene and its derivatives.^[^ ^21^ ^]^ Previous study has successfully in situ formed TiO_2_ nanoparticles on the exterior surface of Ti_3_C_2_ MXene by a hydrothermal method, and the Ti_3_C_2_@TiO_2_ hybrid displayed an excellent reversible capacity in lithium ion batteries (302 mA h g^−1^ at 200 mA g^−1^ after 500 cycles).^[^ ^22^ ^]^ This excellent electrochemical property benefits from the synergy of Ti_3_C_2_ and TiO_2_. However, a significant majority of studies synthesized derived MXene composites through hydrothermal or solvothermal treatment, which required a high temperature or an additional titanium source.^[^ ^23^ ^]^ It is necessary to develop a facile and manageable method to synthesize partially derived MXene hybrids with well‐designed structures for CDI application. As far as we know, there is no report on partially derived MXene materials as CDI electrodes to date.

Herein, a facile one‐step alkalized treatment method was applied to partially derivatize Ti_3_C_2_ MXene in situ to fabricate a hierarchical 1D/2D TiO_2_/Ti_3_C_2_ heterostructure as a cathode material for CDI. Specifically, the cross‐linked 1D TiO_2_ nanowires grown in situ on the surface of 2D Ti_3_C_2_ nanosheets can not only further enlarge the spacing of MXene layers and provide abundant ion transport channels for desalination but also work as a protective layer to alleviate the oxidation of inner Ti_3_C_2_ and thus improve the cycle stability of TiO_2_/Ti_3_C_2_ composites. Moreover, the derived TiO_2_ nanowires can supply additional active sites for high Na^+^ adsorption. The inner Ti_3_C_2_‐preserving 2D framework and outstanding conductivity can facilitate the invertible transmission of electrons and enhance the conductivity of TiO_2_. Meanwhile, the 1D channel of TiO_2_ nanowires can offer fast charge transport paths. Density functional theory (DFT) calculations demonstrate that the generation of TiO_2_‐Ti_3_C_2_ heterostructures accelerates electrons transfer at the interface, which is favorable for promoting electrochemical activity. Profiting from the specific microstructure of 1D/2D hierarchical structure and synergistic effect of TiO_2_ and Ti_3_C_2_, the TiO_2_/Ti_3_C_2_ electrode demonstrates favorable hybrid CDI performance, with a superior desalination capacity of 75.62 mg g^−1^, satisfactory cycling stability, and low energy consumption, which is better than the majority of published MXene‐based electrode materials. And the as‐prepared TiO_2_/Ti_3_C_2_ composites also exhibited superior desalination potential in an ultrahigh NaCl concentration of 500 × 10^−3^ m (173.52 mg g^−1^) which suggested it is promising for seawater treatment. This work provides an effective method for solving the aggregation and oxidation issues of 2D MXene and exploring surface partially derived MXene‐based electrode materials for CDI applications.

## 2 Results and Discussion

### 2.1 Material Characterization

**Figure 1:**
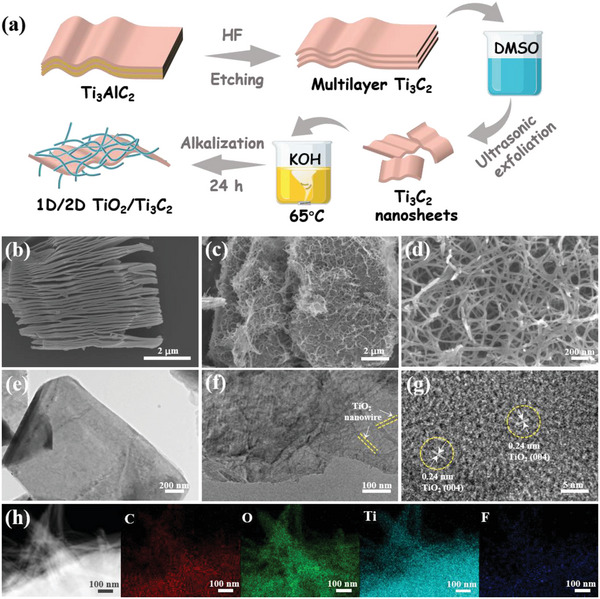
a) Schematic diagram for the preparation of TiO_2_/Ti_3_C_2_; SEM images of b) multilayer Ti_3_C_2_ and c,d) TiO_2_/Ti_3_C_2_; TEM images of e) Ti_3_C_2_ nanosheets and f) TiO_2_/Ti_3_C_2_; g) HRTEM image of TiO_2_/Ti_3_C_2_; h) dark‐filed image of the TiO_2_/Ti_3_C_2_ composite and the corresponding element mapping.

The diagram in **Figure** **1**a illustrates the production process of the hierarchical 1D/2D TiO_2_/Ti_3_C_2_ heterostructure composite (TiO_2_/Ti_3_C_2_). Multilayer Ti_3_C_2_ terminated by ‐OH, ‐O, and ‐F surface groups was prepared via hydrofluoric acid (HF) etching of the bulk precursor Ti_3_AlC_2_ and exhibited a typical accordion sheet‐like structure (Figure 1b), suggesting that the Al layer was successfully removed. Ti_3_C_2_ nanosheets (Figure 1e) were ultimately obtained after dimethyl sulfoxide (DMSO) intercalation and ultrasonic exfoliation. Subsequently, TiO_2_/Ti_3_C_2_ was synthesized through the heat treatment of Ti_3_C_2_ MXene nanosheets in KOH aqueous solution with vigorous stirring. The surface exposed Ti of Ti_3_C_2_ was first attacked by dissolved oxygen to generate TiO_2_ nanoparticles, which acted as nucleation sites. The passive TiO_2_ nanoparticles partially dissolved due to the corrosive action of KOH and underwent a dissolution/recrystallization process to form nanowires.

The concentration of KOH was an important parameter affecting the quantity and morphology of the TiO_2_ nanowires. Figure S2 in the Supporting Information displays the scanning electron microscope (SEM) images of TiO_2_/Ti_3_C_2_ prepared under 8 m KOH (TiO_2_/Ti_3_C_2_‐8). Only a few TiO_2_ nanowires heterogenously grew at the surface and edges of Ti_3_C_2_ MXene with no network of cross‐linked structures. As the concentration of KOH solution increased to 10 m, a large number of TiO_2_ nanowires ≈10–30 nm in diameter were in situ homogenously grown on the lamellar surface (Figure 1c,d). An increase in KOH concentration is favorable for generating thicker nanowires and cohesive filamentous networks. Vigorous stirring also ensured the uniform formation of slender nanowires, and the formed nanowires were bent because of the difference in force applied to the nanowires during agitation.^[^ ^24^ ^]^ The transmission electron microscope (TEM) image of TiO_2_/Ti_3_C_2_ (Figure 1f) showed that a certain number of nanowires were distributed on the flake layer, corresponding to the nanowire/nanosheet composite structure; the Ti_3_C_2_ nanosheets served as both a support and titanium source during the oxidation process. High‐resolution TEM (HRTEM) characterization analysis (Figure 1g) revealed a clear lattice fringe with a lattice spacing of 0.240 nm, representing the (004) crystal plane of anatase TiO_2_. Figure 1h presents the dark‐field TEM images of TiO_2_/Ti_3_C_2_ and its corresponding elemental mappings. It exhibited a uniform distribution of C, Ti, and O elements, demonstrating that TiO_2_ grew uniformly in situ on Ti_3_C_2_. The atomic ratios of C, O, Ti, and F were 12.90%, 38.68%, 40.84%, and 7.58%, respectively (Table S1, Supporting Information). The heterostructure of TiO_2_ and Ti_3_C_2_ was also demonstrated by the selected area electron diffraction speckles (Figure S3, Supporting Information), which exhibited a set of hexagonal diffraction spots and a concentric diffraction ring with a bright center. The hexagonal diffraction spots represent the Ti_3_C_2_ phase, while the diffraction ring represents the reflections from polycrystalline anatase TiO_2_, indicating the coexistence of Ti_3_C_2_ and TiO_2_.^[^ ^25^ ^]^

**Figure 2:**
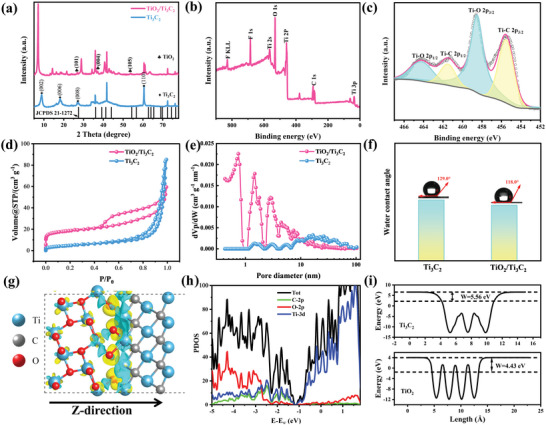
a) XRD pattern of Ti_3_C_2_ and TiO_2_/Ti_3_C_2_; b) full XPS spectrum of TiO_2_/Ti_3_C_2_; c) XPS spectrum of Ti 2p for TiO_2_/Ti_3_C_2_; d) nitrogen adsorption and desorption isotherm of Ti_3_C_2_ and TiO_2_/Ti_3_C_2_; e) pore size distribution of Ti_3_C_2_ and TiO_2_/Ti_3_C_2_; f) water contact angles of Ti_3_C_2_ and TiO_2_/Ti_3_C_2_; g) charge density difference along the *Z* direction for TiO_2_/Ti_3_C_2_; h) DOS analysis of TiO_2_/Ti_3_C_2_; i) theoretically calculated work function of Ti_3_C_2_ and TiO_2_.

**Figure** **2**a depicts the X‐ray diffraction (XRD) curves of Ti_3_C_2_ and TiO_2_/Ti_3_C_2_. The characteristic diffraction peaks of (002), (006), (008), and (110) were observed in the pattern of Ti_3_C_2_, which were highly consistent with those reported for Ti_3_C_2_ MXene.^[^ ^11^ ^]^ New characteristic peaks were observed at 2*θ* = 26.96°, 37.14°, and 54.32° in the curve of TiO_2_/Ti_3_C_2_, which represented the (101), (004), and (105) planes, respectively, of anatase TiO_2_ (JCPDS 21–1271).^[^ ^24^, ^25^ ^]^ The characteristic (002) peak of Ti_3_C_2_ shifted from 7.5° to 7.05° for TiO_2_/Ti_3_C_2_, which corresponds to interlayer distances of 11.77 and 12.52 Å, respectively, indicating a further expansion of interlayer spacing.^[^ ^28^ ^]^ X‐ray photoelectron spectroscopy (XPS) spectra were used to explore the surface chemical composition of the composites. The signals of Ti 2p, C 1s, O 1s, and F 1s were found in the survey spectrum of TiO_2_/Ti_3_C_2_ (Figure 2b). Compared with that of the original Ti_3_C_2_ MXene (Figure S4a, Supporting Information), the atomic content of F substantially decreased, which was due to the substitution of ‐F groups by ‐O or ‐OH groups through the alkali process, and the replacement of F contributed to promoting the specific capacitance.^[^ ^29^ ^]^ The high‐resolution XPS spectrum of Ti 2p for virgin Ti_3_C_2_ MXene (Figure S4b, Supporting Information) demonstrated that the Ti 2p_3/2_ components located at 454.6, 455.5, and 458.7 eV corresponded to Ti‐C, Ti‐X (a combination of a nanostoichiometric TiC*_x_* (*x* < 1)), and TiO_2_, respectively.^[^ ^30^ ^]^ After alkali treatment, the Ti‐X peak at 455.5 eV disappeared, with a decrease in Ti‐C peak intensity and an increase in Ti(IV) peak intensity (Figure 2c), indicating that TiO_2_ was generated by the partial destruction of Ti_3_C_2_.^[^ ^31^ ^]^

The N_2_ adsorption–desorption isotherms of the TiO_2_/Ti_3_C_2_ hybrids displayed a representative type‐IV curve with an H3‐type hysteresis loop (Figure 2d), indicating the predominant mesoporous structures of the compounds and showing a slit pore geometry due to the stacking of spark particles.^[^ ^32^ ^]^ The SSA of TiO_2_/Ti_3_C_2_ (50.74 m^2^ g^−1^) was much larger than that of the pristine MXene nanosheets (*S*_BET_ = 17.56 m^2^ g^−1^) due to the growth of TiO_2_ nanowires on the interlayer increasing the interlayer space of Ti_3_C_2_. Figure 2e displays the pore size distributions of Ti_3_C_2_ and TiO_2_/Ti_3_C_2_, indicating that more mesopores appeared in the TiO_2_/Ti_3_C_2_ structure, which was due to the construction of the 1D/2D hierarchical structure. The larger SSA and abundant mesopores provided more active sites and diffusion paths for ion adsorption and transport during the CDI process.

The above analysis shows that the TiO_2_/Ti_3_C_2_ hybrids with uniform morphology, high purity, and no obvious impurity phase were successfully synthesized. The water contact angle (WCA) measurements were used to explore the wettability of the materials, as shown in Figure 2f. The WCAs of Ti_3_C_2_ and 1D/2D TiO_2_/Ti_3_C_2_ were 129° and 118°, respectively, indicating that the TiO_2_/Ti_3_C_2_ electrode has better wettability, which is beneficial for ion transport during the desalination process.^[^ ^27^ ^]^ After alkali treatment, more ‐OH groups replaced the ‐F groups on the surface of Ti_3_C_2_, which strengthened hydrogen bond formation with H_2_O. Moreover, the 1D/2D hierarchical structure supplies more exposed spaces for electrolyte permeation.

DFT calculations were applied to discuss the diffusion behaviors at the interface of the heterostructures. The optimized crystal structure of the TiO_2_/Ti_3_C_2_ heterostructure (Figure S5a, Supporting Information) demonstrated the formation of the Ti—O band between the TiO_2_ and Ti_3_C_2_, and the length of the Ti—O band at the interface was 1.98 Å, which is approximately equal to the actual length of the Ti—O band (1.94–2.01 Å) in pristine TiO_2_.^[^ ^33^ ^]^ Interfacial interactions were studied by analyzing the charge density differences. The yellow and cyan areas in Figure 2g refer to electron accumulation and depletion, respectively. Electronic coupling occurred at the interface between the TiO_2_ and Ti_3_C_2_ surfaces, and Ti_3_C_2_ contributed electrons, while TiO_2_ acquired electrons at the Ti_3_C_2_/TiO_2_ heterostructure interface. The planar electrostatic potential of TiO_2_/Ti_3_C_2_ along the *Z*‐direction further demonstrated the electron transport process (Figure S5b, Supporting Information). Figure S5c,d in the Supporting Information presents the densities of states (DOSs) of TiO_2_ and Ti_3_C_2_, respectively. The energy gap of TiO_2_ near the Fermi level was ≈1.5 eV, demonstrating the semiconductor properties of TiO_2_. The valence bands of Ti_3_C_2_ across the Fermi level are indicative of its metallic characteristics. Compared to TiO_2_ and Ti_3_C_2_, there were quite a few electronic states across the Fermi level in the TiO_2_/Ti_3_C_2_ heterostructure (Figure 2h), suggesting that the electrical conductivity of TiO_2_ was notably improved. The work function describes the electron transfer on the heterojunction interface of TiO_2_/Ti_3_C_2_. The work functions of Ti_3_C_2_ and TiO_2_ (Figure 2i) were 4.43 and 5.56 eV, respectively, implying that Ti_3_C_2_ tends to continuously inject electrons into TiO_2_ to maintain the charge balance, which will significantly improve the electrochemical performance and facilitate the kinetic process of the TiO_2_/Ti_3_C_2_ heterostructure.

### 2.2 Electrochemical Performance

**Figure 3:**
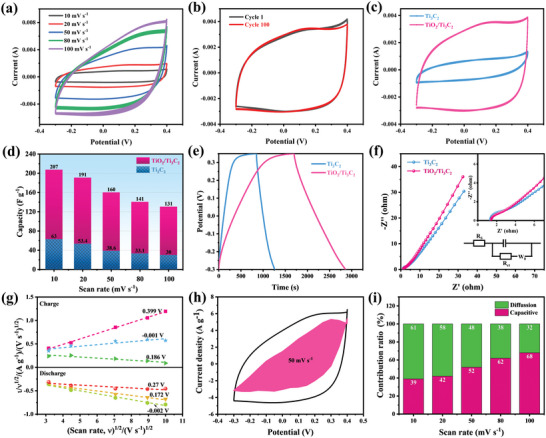
a) CV curves of TiO_2_/Ti_3_C_2_ at various scan rates; b) CV curves over 100 cycles at a scan rate of 50 mV s^−1^; c) CV curves of TiO_2_/Ti_3_C_2_ and TiO_2_ at a scan rate of 50 mV s^−1^; d) comparison of capacitance between TiO_2_/Ti_3_C_2_ and Ti_3_C_2_; e) GCD curves of TiO_2_/Ti_3_C_2_ and Ti_3_C_2_ at 0.1 A g^−1^; f) Nyquist plots of TiO_2_/Ti_3_C_2_ and Ti_3_C_2_; g) plots of *i*/*ν* ^1/2^ versus *ν* ^1/2^ at various potentials; h) CV curve at 50 mV s^−1^ where the shaded area represents capacitive contribution; i) capacitive and diffusion contribution ratio at various scan rates.

The cyclic voltammetry (CV) curves of TiO_2_/Ti_3_C_2_ at various scan rates presented an equirectangular shape without observable redox peaks (**Figure** **3**a), proving that the composites exhibited Faradaic pseudocapacitive behaviors with a larger capacity. The slight losses in capacity at high scan rates suggested a rapid charge storage principle. TiO_2_/Ti_3_C_2_ also showed excellent cyclic stability, followed by the shapes of the CV curves barely changing after going through 100 cycles, and the specific capacity retention rate was 100.9% (Figure 3b). The capacitance slightly increased after a long cycle, which might be attributed to the contact between the TiO_2_ nanowires and Ti_3_C_2_ nanosheets becoming tighter due to the electroactivation effect during the ion deintercalation cycle, further improving the electrochemical performance of TiO_2_/Ti_3_C_2_.^[^ ^34^ ^]^ Figure 3c compares the CV curves of TiO_2_/Ti_3_C_2_ and the initial Ti_3_C_2_ at 50 mV s^−1^, and the specific capacitances under different specific currents are displayed in Figure 3d. The specific capacitance of TiO_2_/Ti_3_C_2_ was 207 F g^−1^ at 10 mV s^−1^, while that of Ti_3_C_2_ was only 63 F g^−1^. To thoroughly compare the electrochemical performance of as‐prepared TiO_2_/Ti_3_C_2_ with that of previously developed MXene‐based electrodes, we measured the electrochemical capacity of the TiO_2_/Ti_3_C_2_ heterostructure in different electrolytes (1 m H_2_SO_4_, 1 m Li_2_SO_4_, 1 m Na_2_SO_4_, and 1 m KOH) using a three‐electrode cell configuration (Figure S6a–d, Supporting Information). To estimate the Li^+^ storage capacity of TiO_2_/Ti_3_C_2_, we also measured its CV profiles in 1 m LiPF_6_ with a mixture of ethylene carbonate and dimethyl carbonate (1:1 by volume) (Figure S6e, Supporting Information). Table S2 in the Supporting Information lists the capacitance values for the reported state‐of‐the‐art MXene‐based heterostructure electrodes in different electrolytes. Remarkably, our work achieved a relatively high specific capacity, which is comparable to or even higher than that of currently developed MXene‐based electrodes. The significantly improved specific capacitances were closely related to the increased interlayer spacing and unique 1D/2D nanowire/nanosheet hierarchical structure, which provides more ion adsorption sites.

The galvanostatic charge–discharge (GCD) curves without observable redox peaks exhibited the pseudocapacitive properties of TiO_2_/Ti_3_C_2_ and Ti_3_C_2_ (Figure 3e), which were consistent with the conclusion of the CV curves. In addition, the charging/discharging process was asymmetric, implying that the ion capture behavior of TiO_2_/Ti_3_C_2_ can be categorized as pseudocapacitive behavior rather than electrical double‐layer behavior. The Nyquist plots of TiO_2_/Ti_3_C_2_ and Ti_3_C_2_ (Figure 3f) fitted by the equivalent circuit model showed a semicircle (Figure 3f inset) and a straight line at low frequency. The charge transfer resistance (*R*_ct_) values estimated from the semicircle for Ti_3_C_2_ and TiO_2_/Ti_3_C_2_ (Table S3, Supporting Information) were 1.25 and 0.81 Ω, respectively. TiO_2_/Ti_3_C_2_ displayed a lower charge transfer resistance, which might be because of the high electrical conductivity of MXene and the abundant ion transmission channels in the TiO_2_/Ti_3_C_2_ hybrid. The electrochemical impedance spectra (EIS) analysis demonstrated that TiO_2_/Ti_3_C_2_ with superior electrical conductivity was conducive to the rapid transport of ions and had an excellent electrochemical performance.^[^ ^35^ ^]^

To explore the superior rate performance of the TiO_2_/Ti_3_C_2_ electrode in depth, the proportion of surface‐controlled capacitance (capacitor‐like contribution) was analyzed (Figure 3g–i).^[^ ^36^ ^]^ A dominant capacitive contribution was realized for the TiO_2_/Ti_3_C_2_ electrode (52% at 50 mV s^−1^). The percentage of capacitive contribution increased with increasing scan rate, reaching a maximum of 68% at 100 mV s^−1^ (Figure 3i). Notably, a large capacitance contributes to facilitating fast and reversible ion storage.^[^ ^37^ ^]^

### 2.3 Desalination Performance

**Figure 4:**
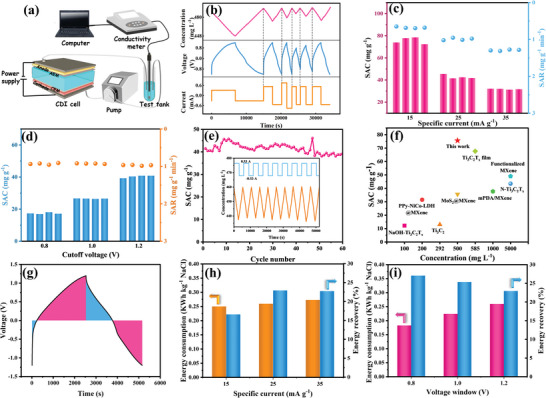
a) Schematic diagram of the CDI system; b) changes in the NaCl concentration, voltage, and current over time under various current densities and voltage windows; salt adsorption capacity and rate of TiO_2_/Ti_3_C_2_ c) under different current densities and d) voltage windows; e) Long‐term desalination capacity and the corresponding change in salt concentration over time; f) comparison of the desalination performance of MXene‐based composites in NaCl solutions with different concentrations; g) change in voltage over time during the desalination/regeneration process; energy consumption and energy recovery under h) different current densities and i) voltage windows.

A hybrid CDI (HCDI) cell equipped with dual ion‐exchange membranes (**Figure** **4**a) was used to test the desalination performance of TiO_2_/Ti_3_C_2_ at a specific current. The variations in the NaCl concentration, voltage, and current over time under different current densities and cutoff voltages that occurred during the electrosorption/regeneration process are presented in Figure 4b. The NaCl concentration varied linearly with the reaction time and could be restored to its original state, which demonstrated the excellent regeneration performance of the electrode.^[^ ^38^ ^]^ Moreover, the NaCl concentration decreased during the charging process while increasing during the discharging process, indicating that the adsorption/desorption reactions of Na^+^/Cl^−^ occurred during the charging/discharging process. The salt adsorption capacity (SAC) was 75.62 mg g^−1^ at 15 mA g^−1^ and declined to 31.71 mg g^−1^ as the specific current increased to 35 mA g^−1^ (Figure 4c). A large current density contributes to a shorter charging time and large diffusion restriction; thus, abundant active sites are underutilized, resulting in a reduced capacity, while a larger current density can accelerate charge transfer, giving rise to a faster desalination rate. When the current density was 35 mA g^−1^, the specific absorption rate (SAR) reached 1.30 mg g^−1^ min^−1^. When the voltage window increased from −0.8 V/0.8 V to −1.2 V/1.2 V, the average SAC increased from 17.35 to 42.78 mg g^−1^ A larger voltage range with a longer charging time accumulates more charge at the electrode, which is beneficial for the CDI process. Nevertheless, an overly high cutoff voltage is prone to inducing side reactions and reduces the stability of electrodes.^[^ ^39^ ^]^ The developed TiO_2_/Ti_3_C_2_ electrode in the membrane‐free CDI system also exhibited an exceptional desalination capacity (53.3 mg g^−1^ at 15 mA g^−1^), as shown in Figure S7 in the Supporting Information.

The cycle stability of electrodes is a significant index for judging their CDI properties. The SAC of the TiO_2_/Ti_3_C_2_ electrode remained at a capacity retention ratio of 94% after 60 cycles at a current density of 25 mA g^−1^ (Figure 4e), and even after 200 cycles, the TiO_2_/Ti_3_C_2_ electrode still had a good capacity retention of 71% (Figure S8, Supporting Information), suggesting a preferable desalination cyclicity. The cycle stability under a low current density (15 mA g^−1^) was also analyzed because the desalination time is longer at lower current densities. The TiO_2_/Ti_3_C_2_ electrode exhibited a good capacity retention ratio of 87% after 40 cycles at 15 mA g^−1^ (Figure S9, Supporting Information), indicating its desalination stability at low current densities. The excellent cycle stability is enhanced by the protection of the wrapped TiO_2_ nanowire layer on the surface of the Ti_3_C_2_ sheet, which delays the oxidation of the inner Ti_3_C_2_ and improves the stability of the TiO_2_/Ti_3_C_2_ composites.

The CDI properties of various MXene‐based electrodes are summarized in Figure 4f and Table S4 in the Supporting Information. To facilitate a comparison with previous reports, the CDI performance was also tested under a constant applied voltage (64.32 mg g^−1^ at 1.2 V over 1 h, Figure S10, Supporting Information). The TiO_2_/Ti_3_C_2_ electrode in this work exhibited an optimum desalination capacity and excellent CDI performance, which was due to the large SSA, abundance of active sites, and short ion diffusion pathway provided by the unique 1D/2D hierarchical structure and good electrochemical performance of the TiO_2_/Ti_3_C_2_ heterostructure. In addition, the desalination properties of the as‐prepared TiO_2_/Ti_3_C_2_ electrode in an ultrahigh NaCl concentration (500 × 10^−3^ m) were analyzed (Figure S11, Supporting Information), and it displayed superior CDI performance (173.52 mg g^−1^ at an operation voltage of 1.2 V for 2 h), suggesting its promising potential for seawater desalination.

Specific energy consumption (SEC) and charge efficiency (CE) are incredibly important for the practical application of CDI. Faradaic materials display less energy consumption per ion adsorption than traditional carbon materials.^[^ ^40^ ^]^ The total energy consumption during a typical charging/discharging process was obtained from the voltage–time curve (Figure 4g). The blue part represents SEC, and the pink part represents energy recovery. The energy recovery rate (%) was obtained by the ratio of the recovered energy to the consumed energy. First, a self‐discharging process occurred under voltages of −1.2 to 0 V; then, the voltage increased from 0 to 1.2 V, causing significant ion intercalation into the electrode materials. When the voltage decreased from 1.2 to 0 V, ions were observably deintercalated from the electrodes and released into the salt solution in the last step.^[^ ^41^ ^]^ Figure 4h,i distinguishes the energy consumption and recovery at different current densities. The SEC was within 0.14–0.41 kWh kg^−1^‐NaCl and 0.13–0.20 kWh kg^−1^‐NaCl at different current densities and various voltage windows, respectively, which was much lower than that of typical carbonaceous materials (i.e., AC with 1.11 kWh kg^−1^‐NaCl). To facilitate a comparison with previous studies, the energy consumption required for treating 1 L feed water (*E*_V_, Wh m^−3^) is also presented in Figure S12 in the Supporting Information. The *E*_V_ was as low as 0.96 Wh m^−3^, which is much lower than that of typical MXene‐based electrodes (Table S4, Supporting Information). The reduction in current density and increase in cutoff voltage result in more energy consumption, and the voltage window exhibits an influence on SEC. A large voltage window easily causes side reactions, such as the reduction of dissolved oxygen, resulting in a lower energy recovery rate. TiO_2_/Ti_3_C_2_ displayed a high CE of 92.81% at a current density of 15 mA g^−1^ (Figure S13, Supporting Information). The relatively high CE is attributed to the low charge transfer resistance of TiO_2_/Ti_3_C_2_, which improves the charge transfer efficiency. Figure S14 in the Supporting Information depicts the ion storage process in the HCDI cell.

The excellent desalination performance of asymmetric AC//TiO_2_/Ti_3_C_2_ is ascribed to the assembly of 1D/2D heterostructures and TiO_2_/Ti_3_C_2_ heterostructures, which display significant SSA, abundant pore structure, and excellent electrochemical properties, leading to a large desalination capacity and fast desalination rate. In addition, the TiO_2_ coating partially derived from MXene can work as a protective layer to alleviate the oxidation of the remaining Ti_3_C_2_, which contributes to maintaining the excellent cycling stability of TiO_2_/Ti_3_C_2_ electrodes.

## 3 Conclusion

In this study, we prepared a well‐designed hierarchical TiO_2_/Ti_3_C_2_ heterostructure by partially derivatizing Ti_3_C_2_ MXene in situ. The conductive 2D Ti_3_C_2_ nanosheets bridged the 1D TiO_2_ nanowires to fabricate a 1D/2D hierarchical heterostructure. This unusual morphology not only provides abundant active sites due to the large SSA but also guarantees excellent electrochemical properties under the synergistic effect of TiO_2_ and Ti_3_C_2_. The obtained TiO_2_/Ti_3_C_2_ composite exhibits superior HCDI performance, with a maximum SAC of 75.62 mg g^−1^, maximum SAR of 1.3 mg g^−1^ min^−1^, and prominent cyclic stability (without a distinct downtrend after 60 cycles). Remarkably, the desalination performance of TiO_2_/Ti_3_C_2_ is significantly better than that of the majority of published MXene‐based materials, revealing the promising application prospects of hierarchical TiO_2_/Ti_3_C_2_ heterostructures in the field of CDI.

## 4 Experimental Section

### Synthesis of Ti_3_C_2_ MXene and 1D/2D TiO_2_/Ti_3_C_2_

Ti_3_AlC_2_ powders (1.0 g, 400 mesh, 11 Technology Co., Ltd.) were smoothly dispersed into 20 mL of HF (Aladdin Industrial) at 30 °C with constant stirring for 72 h at 300 rpm to etch the Al layer from Ti_3_AlC_2_. The sediment was obtained by centrifugation and washed successively with deionized (DI) water and ethanol (Aladdin Industrial) until the suspension pH was higher than 6, and Ti_3_C_2_ was obtained after freeze‐drying. The as‐prepared Ti_3_C_2_ (0.6 g) was mixed with 10 mL of DMSO (Sinopharm Chemical Reagent Co., Ltd.) under stirring for 24 h at room temperature. The synthetic material was cleaned with DI water four times by centrifugation. After that, the mixture underwent ultrasonic treatment for 1 h under argon protection and then the sediment was separated by centrifugation at 3500 rpm for 10 min. The monolayer Ti_3_C_2_ was obtained by supernatant freeze‐drying. A total of 0.6 g of the monolayer Ti_3_C_2_ prepared above was added to a 10 m KOH solution (Sinopharm Chemical Reagent Co., Ltd.) at 65 °C accompanied by stirring for 24 h at 350 rpm. Wash the precipitates with DI water until the pH of supernatant reaches 7. After centrifugation, the samples were freeze‐dried to obtain TiO_2_/Ti_3_C_2_ composites. All chemicals and reagents used in this work were used without further purification.

### Electrode Preparation

The electrode was prepared as follows: active material (Ti_3_C_2_, TiO_2_/Ti_3_C_2_, or active carbon), acetylene black (Sinopharm Chemical Reagent Co., Ltd.), and polyvinylidene fluoride (Shanghai Macklin Biochemical Co., Ltd.) at a weight ratio of 80%:10%:10% were blended in a specified volume of *N*‐methyl‐2‐pyrrolidone (Aladdin Industrial) solvent under stirring for 12 h to obtain a homogenous slurry. Then, the as‐prepared slurry was uniformly dropped on graphite paper using a doctor‐blade method with a thickness of ≈75 µm. The thin film electrodes were vacuum‐dried at 60 °C for 12 h.

### Material Characterization

The microstructure of the obtained samples was observed using SEM (Hitachi S‐4800/EX‐350, Japan), TEM (JEOL‐2010F, Japan), and HRTEM (JEM‐2010F, Japan). XRD patterns using nickel‐filtered Cu K*α* radiation (*λ* = 1.5406 Å) at 40 kV and 40 mA (Bruker D8 Advance, Germany) were obtained to research the crystalline phase of the samples. XPS was performed using a Kratos Axis Ultra DLD spectrometer (XSAM 800 spectrometer, Kratos Co., UK) with a monochromated Al K*α* X‐ray source (energy 1486.68 eV) to analyze the surface composition of the materials. The SSA of the samples was measured by a BELSORP instrument (BEL, Japan, Inc.) on the basis of N_2_ adsorption/desorption isotherms acquired at 77 K by Brunauer–Emmett–Teller (BET) methods. The hydrophilicity of the samples was studied by the WCA test using an optical contact angle measurement system (POWEREACH JC2000, China).

### DFT Calculation

The first‐principles approach based on DFT was applied to simulate the mutual effect between different components using the Vienna Ab initio Simulation Package (VASP).^[^ ^42^ ^]^ The Perdew–Burke–Ernzerhof exchange‐correlation functional in generalized gradient approximation was used for molecular geometry optimization and total energy computation.^[^ ^43^ ^]^ To isolate the interactions between each sheet, a 15 Å vacuum was introduced. The cutoff energy of a plane wave base was set as 520 eV to optimize the structure. Brillouin zone integrations were set up with a 3 × 2 × 1 gamma‐centered *k*‐point for static computations. For all organized geometric optimization structures, the energy convergence accuracy was 1.0 × 10^−6^ eV, and the force tolerance for geometry optimization was 0.03 eV Å^−1^. TiO_2_ (101) and Ti_3_C_2_ (001) sections with specific surface carriers were meticulously designed for lattice matching. The lattice constants of the TiO_2_ (101) model were *u* = 7.552 Å, *v* = 10.21 Å, and *θ* = 90°. Those of the Ti_3_C_2_ (001) model were *u* = 7.13 Å, *v* = 10.61 Å, and *θ* = 90°. The lattice mismatch was less than 5.5%. The TiO_2_ (101) model was consisted of 16 Ti atoms and 32 O atoms. During geometric optimization, the bottom half of these atoms was fixed.

### Electrochemical and Desalination Experiments

The CV, GCD, and EIS tests were conducted using a CHI 660D electrochemical workstation (Shanghai CH Instruments Co., China) in a 1 m NaCl aqueous solution. The electrochemical tests involved a three‐electrode configuration composed of a working electrode, reference electrode (Ag/AgCl electrode), and counter electrode (platinum sheet). Ti_3_C_2_ and TiO_2_/Ti_3_C_2_ films (1 × 1 cm^2^) were directly used as working electrodes for the electrochemical measurements. The CV curves were obtained between a voltage window of −0.3 to 0.4 V under specific scan rates (10–100 mV s^−1^). Then, the GCD was measured within the uniform potential window under various current densities (0.1–1 A g^−1^). The EIS performance was evaluated at a particular frequency range (10^5^ to 10^−2^ Hz) with an amplitude of 5 mV.

Batch‐mode desalination experiments were performed in NaCl aqueous solution under a continuous circulation system composed of a CDI device, conductivity meter (Mettler Toledo S230, Switzerland), peristaltic pump (Longer Pump, YZ‐1515x), constant current power supply equipment (LAND battery testing system), and NaCl solution tank. The flow‐by CDI cell shown in Figure S1 in the Supporting Information was consisted of a cathode (TiO_2_/Ti_3_C_2_) with an area of 4 × 4 cm^2^ and a mass loading of ≈16 mg for the active material, an anode (AC), a cation/anion exchange membrane (CEM/AEM), several glass plates and silicone gaskets, and a chamber with a volume of 0.7 × 5 × 5 cm^3^. All electrodes were prepared according to the abovementioned method. In the course of operation, 40 mL of NaCl aqueous solution (500 mg L^−1^) was constantly cycled from the tank to the CDI apparatus and then pumped back into the tank with a flow velocity of 20 mL min^−1^. A conductivity meter (METTLER TOLEDO S230, Switzerland) was introduced to measure the real‐time electrical conductivity. Desalination experiments were performed under a constant current, and the electrodes were subjected to 12 h of electrolyte immersion and five previous charge/discharge CDI cycles. The data obtained in the sixth cycle were used to calculate the desalination capacity (SAC) and the charge efficiency (CE, *Λ*, %). The effects of different current densities (15, 25, and 35 mA g^−1^) and cutoff voltages (0.8, 1.0, and 1.2 V) on the SAC of the electrodes were investigated. The SAC was calculated using the total mass of the active materials of the TiO_2_/Ti_3_C_2_ electrode. Specific computational formulas are presented in the Supporting Information.

## Conflict of Interest

The authors declare no conflict of interest.

## Supporting information

Supporting Information

## Data Availability

The data that support the findings of this study are available from the corresponding author upon reasonable request.
