# Supplementary material for: Ti3C2‐MXene Partially Derived Hierarchical 1D/2D TiO2/Ti3C2 Heterostructure Electrode for High‐Performance Capacitive Deionization
Source: Adv Sci (Weinh). 2022 Nov 28;10(2):2204041. doi: 10.1002/advs.202204041 (PMC9839853; doi:10.1002/advs.202204041)
Supplement: Supplementary file 1 — Supporting Information [file ADVS-10-2204041-s001.pdf]

ADVANCED SCIENCE
Open Access

Supporting Information

for *Adv. Sci.*, DOI 10.1002/advs.202204041

Ti_3C_2-MXene Partially Derived Hierarchical 1D/2D TiO_2/Ti_3C_2 Heterostructure Electrode for High-Performance Capacitive Deionization

*Ningning Liu, Lanlan Yu, Baojun Liu, Fei Yu, Liqing Li, Yi Xiao, Jinhu Yang and Jie Ma**

Supporting information

Ti_3C_2-MXene Partially Derived Hierarchical 1D/2D TiO_2/Ti_3C_2 Heterostructure Electrode for High-Performance Capacitive Deionization

Ningning Liu[1], Lanlan Yu[2], Baojun Liu[2], Fei Yu[3], Liqing Li[4], Yi Xiao[5], Jinhu Yang[6], Jie Ma[1,4*]

1 Research Center for Environmental Functional Materials, State Key Laboratory of Pollution Control and Resource Reuse, College of Environmental Science and Engineering, Tongji University, 1239 Siping Road, Shanghai 200092, P.R. China

2 College of Resource and Environmental Engineering, Guizhou University, Guiyang, 550025, China

3 College of Marine Ecology and Environment, Shanghai Ocean University, Shanghai 201306, P.R. China

4 Faculty of Materials Metallurgy and Chemistry, Jiangxi University of Science and Technology, Ganzhou, 341000, P.R. China

5 School of Chemical Science and Engineering, Tongji University, 1239 Siping Road, Shanghai, 200092, P. R. China

6 Institute of Materials Science, TU Darmstadt 64287, Darmstadt, Germany

Computational formula

The specific capacity (C) was calculated on the basis of Eq. (1).

$$C = \frac{\int I \times dt}{m \times v \times \Delta V} \quad (1)$$

Where C is the specific capacitance (F g^{-1}), I is the current (A), v is the scan rate (V s^{-1}), ΔV is the applied potential window (V), and m is the electrode material mass (g). CV curves at different scan rates were used to quantify the contributions from diffusion-controlled process ($k_1 v^{1/2}$) and capacitive process ($k_2 v$), according to Eq. (2) and Eq. (3).

$$i_{total} = i_{insertion} + i_{capacitive} = k_1 v^{(1/2)} + k_2 v \quad (2)$$

$$i(V)/v^{(1/2)} = k_1 + k_2 v^{(1/2)} \quad (3)$$

Where $i(V)$ and v represent the total current (A) at a given potential and the sweep rate (v, V s^{-1}) of a CV experiment. k_1 or k_2 is constant.

The SAC (mg g^{-1}) was calculated based on the conversion of conductivity to NaCl concentration, as presented in the following equation:

$$SAC = \frac{(C_0 - C_e) \times V}{m} \quad (4)$$

where C_0 and C_e are the concentration of NaCl at initial and final stages (mg L^{-1}), respectively; V is the volume of NaCl solution (L); and m is the total active mass of the TiO_2/Ti_3C_2 cathode (e.g., 0.016 g).

The SAR (mg g^{-1} min^{-1}) was calculated following to Eq. (5).

$$SAR = \frac{SAC}{t} \quad (5)$$

Where SAC is the desalination capacity (mg·g^{-1}), and t is the desalination time (min). The energy consumption required for removing 1 Kg NaCl (E_M, KWh Kg^{-1} NaCl) and for treating 1 L feed water (E_V, Wh m^{-3}) are calculated by the following formula:

$$E_{in} = \int_0^{t_{cycle}} IVdt, where\ IV > 0 \quad (6)$$

$$E_{out} = \int_0^{t_{cycle}} IVdt\,, where\ IV < 0 \quad (7)$$

$$E_m = \frac{E_{in} - \eta E_{out}}{(C_0 - C_e)V_d} \quad (8)$$

$$E_v = \frac{E_{in} - \eta E_{out}}{V_d} \quad (9)$$

where *IV* is the current-voltage product for a single electrode pair (W), t_{cycel} means the time corresponding to one desalination cycle (min). E_{in} is the total energy input during the batch-mode cycle (J), E_{out} is the total recoverable energy from the cell over the batch mode cycle (J), and η is the fraction of E_{out} actually recovered and re-used to power another charging phase. η is a part of E_{out}, which actually represents the next charging stage of recycling and reuse. Theoretically, the η approximately equals to 1. V_d is the volume of desalinated water collected, the value of V_d in our work is 0.00004 m^3. C_0 and C_e are the concentration of NaCl at initial and final stages, respectively.

Charge efficiency (CE, Λ) of the HCDI system was calculated based on the ratio of the removed salt to charge passed through the cell, according to the following formula:

$$\Lambda = \frac{\Gamma F}{\Sigma} \quad (10)$$

Where Λ is the charge efficiency (%), F is the Faraday constant (96 485 C mol^{-1}), Γ is the total salt adsorption (mol g^{-1}), and Σ is the total charge during the CDI adsorption process (C).

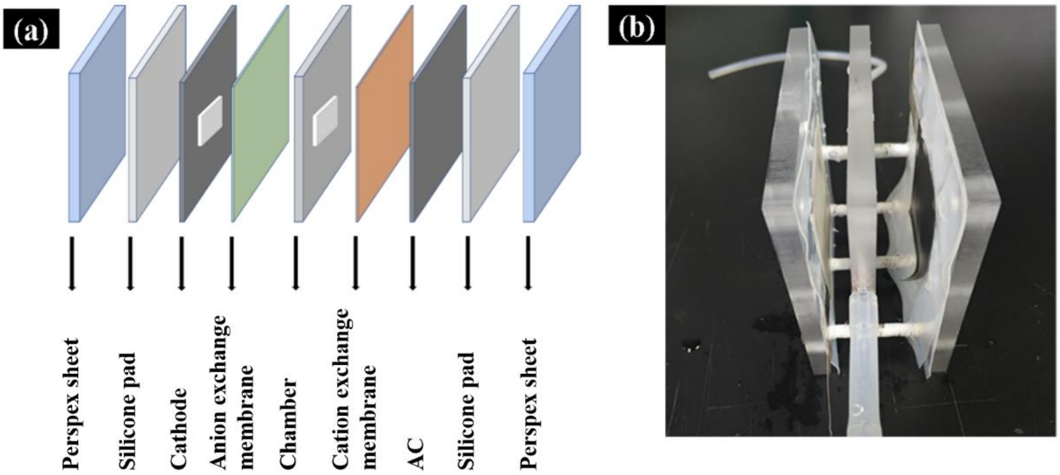

Figure S1 Schematic diagram and image of the CDI unit cell.

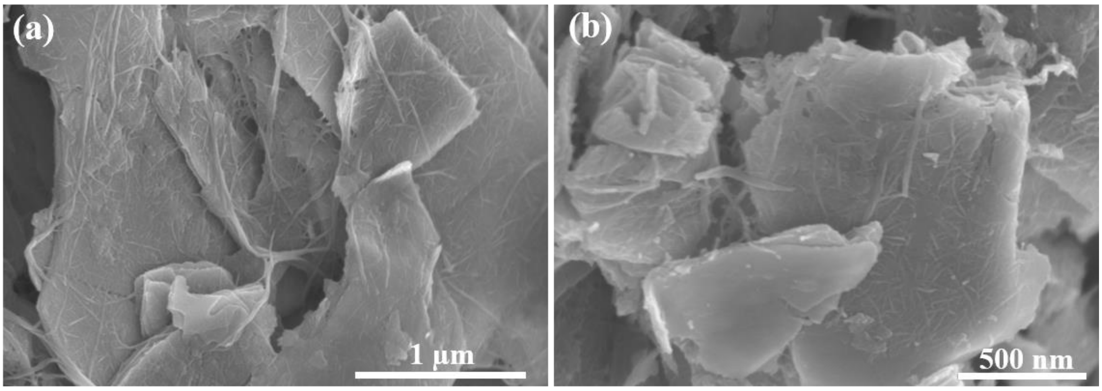

Figure S2 SEM images of TiO_2/Ti_3C_2-8.

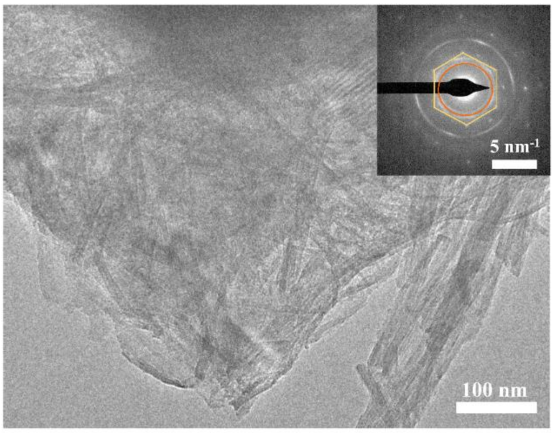

Figure S3 SAED pattern of TiO_2/Ti_3C_2 composite.

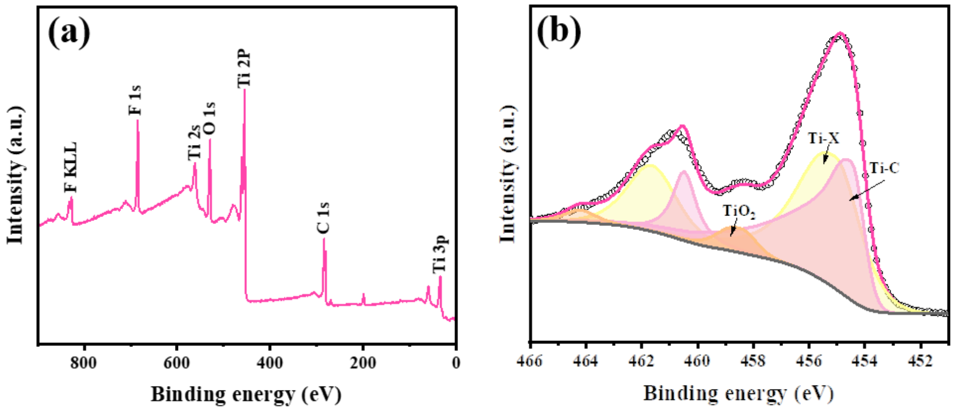

Figure S4 XPS survey spectrum and high-resolution Ti 2p of Ti_3C_2

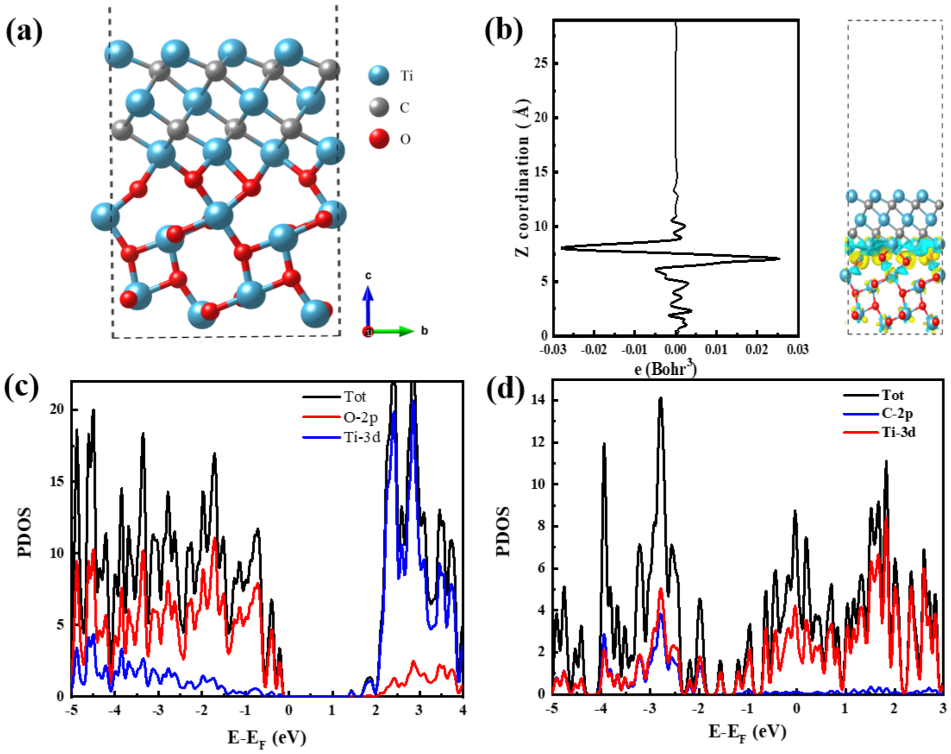

Figure S5 (a) Crystal model of TiO_2/Ti_3C_2 heterojunction after relaxation; (b) Planar electrostatic potential and charge difference of TiO_2/Ti_3C_2; DOS analysis of (c) TiO_2 and (d) Ti_3C_2.

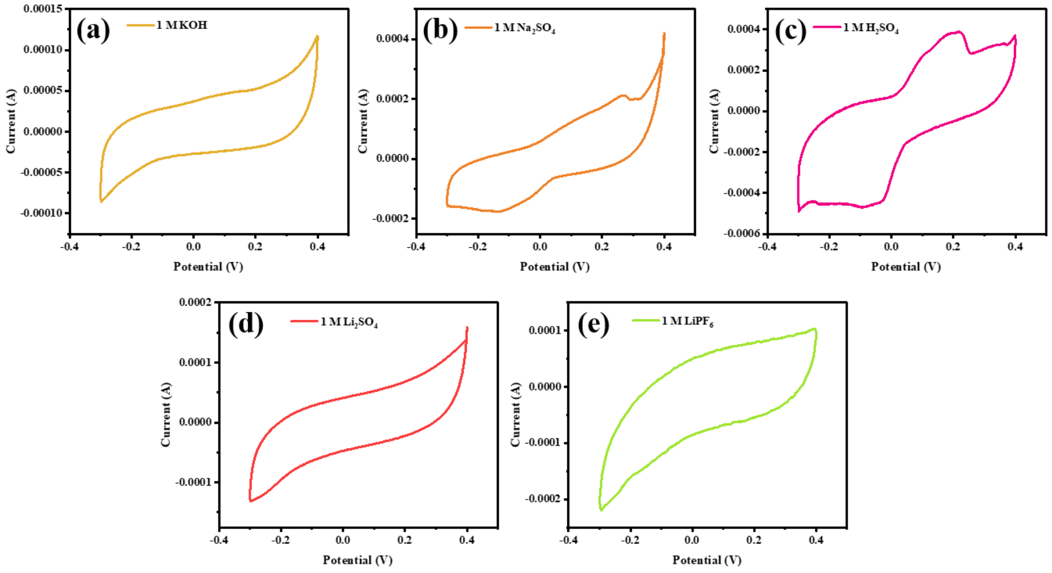

Figure S6 CV profiles of TiO_2/Ti_3C_2 in different electrolytes at scan rate of 1 mV s^{-1}.

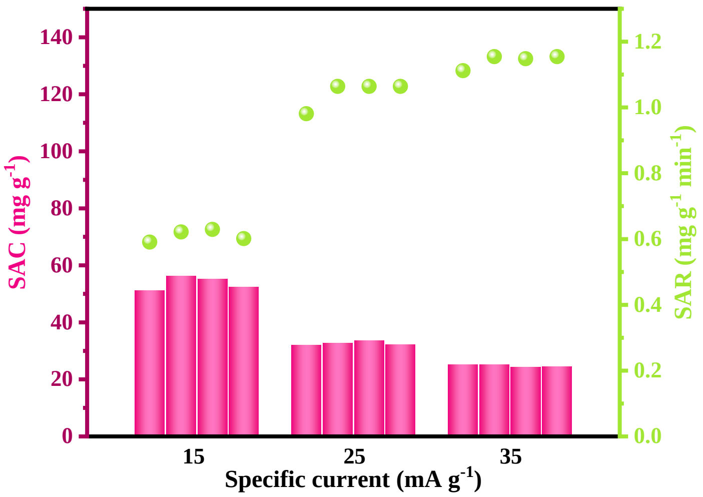

Figure S7 Salt adsorption capacity and rate of TiO_2/Ti_3C_2 under different current densities in membrane-free CDI system.

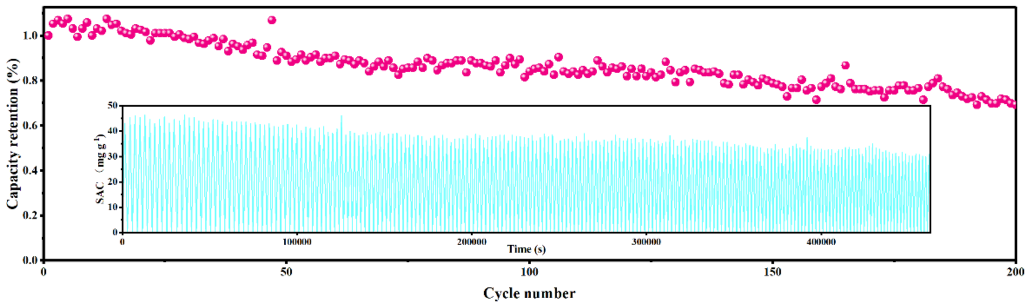

Figure S8 Desalination capacity retention of TiO_2/Ti_3C_2 electrode at the current density of 25 mA g^{-1} over 200 cycles and the corresponding change in desalination capacity over time.

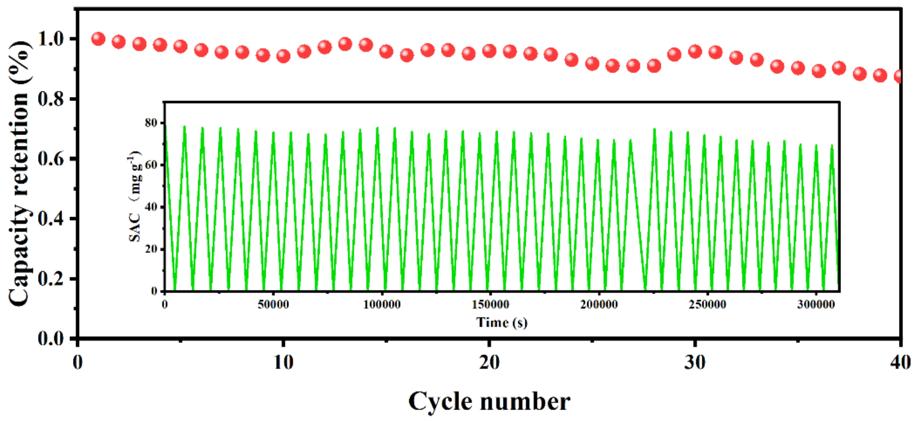

Figure S9 Desalination capacity retention of TiO_2/Ti_3C_2 electrode at the current density of 15 mA g^{-1} over 40 cycles and the corresponding change in desalination capacity over time.

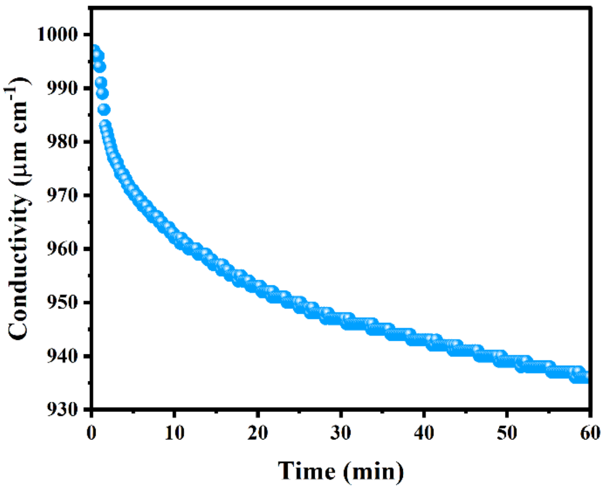

Figure S10 Plot of NaCl solution conductivity variation versus time during the desalination process tested in 500 mg L^{-1} NaCl solution at an applied voltage of 1.2 V for 60 min.

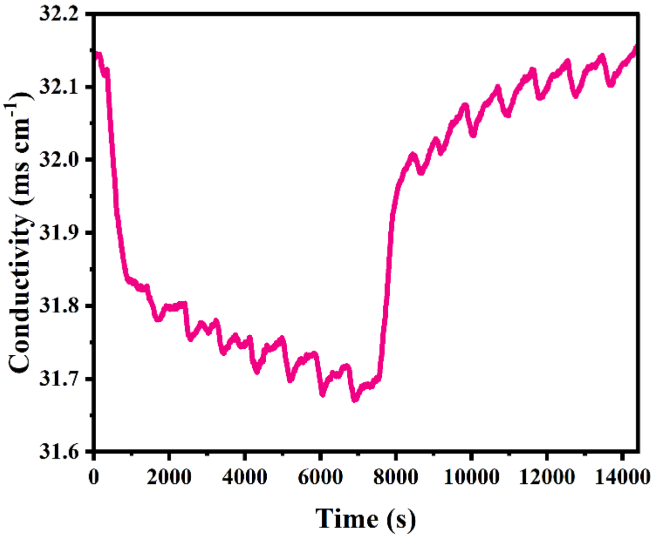

Figure S11 Solution conductivity vs. runtime of TiO_2/Ti_3C_2 electrode in a NaCl aqueous solution of 500 mM upon an external voltage of 1.2 V.

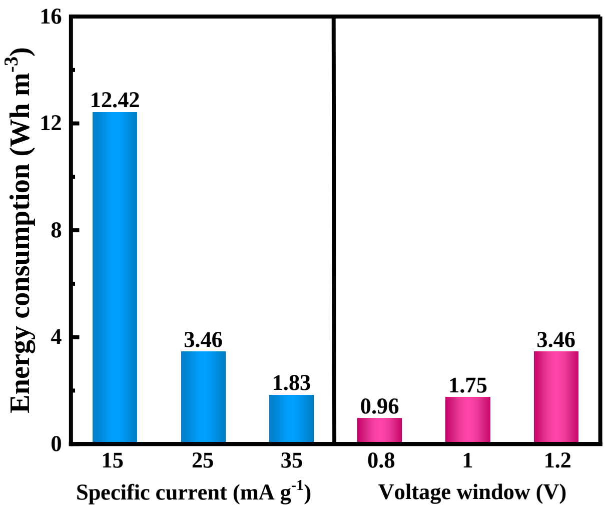

Figure S12 Energy consumption required for treating 1 L feed water (E_V, Wh m^{-3}) at different current density and voltage windows.

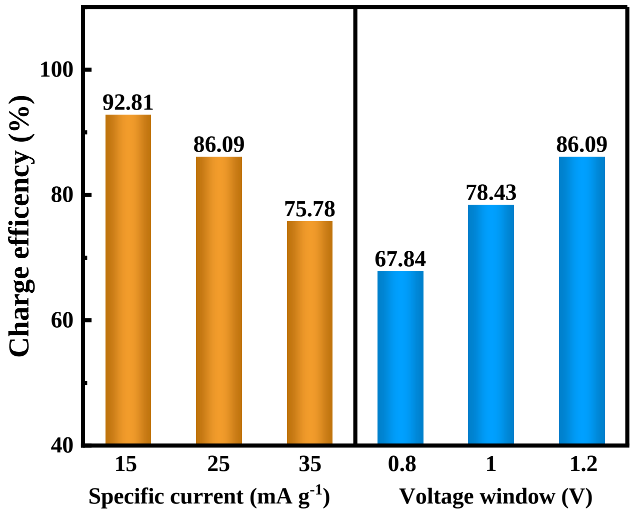

Figure S13 Charge efficiency of TiO_2/Ti_3C_2 under various operating conditions.

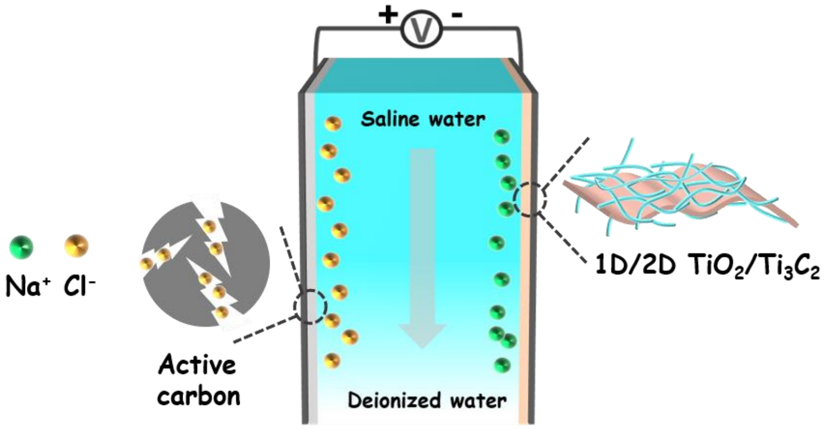

Figure S14 Schematic diagram for the asymmetric CDI cell (AC//TiO_2/Ti_3C_2)

Table S1 Atomic fraction of elements on the surfaces of Ti_3C_2/TiO_2 composites.

Element	Atomic Fraction (%)
C	12.90
Ti	40.84
O	38.68
F	7.58

Table S2 Capacitance comparison with other electrodes based on TiO_2/MXene heterostructures

Electrode materials	Scan rate/ Current density	Electrolyte	Specific capacity	Ref
TiO_2-Ti_3C_2	5 mV s^{-1}	6 M KOH	143 F g^{-1}	[1]
TiO_2/Ti_3C_2	0.3 A g^{-1}	2 M KOH	102.5 F g^{-1}	[2]
TiO_2/$Ti_3C_2T_x$ -350	1 mV s^{-1}	1 M NaCl	164 F g^{-1}	[3]
PANI@TiO_2/$Ti_3C_2T_x$	10 mV s^{-1}	1 M KOH	188.3 F g^{-1}	[4]
GO/TiO_2	5 mV s^{-1}	1 M Na_2SO_4	100 F g^{-1}	[5]
N-$Ti_3C_2T_x$	1 mV s^{-1}	1 M H_2SO_4	192 F g^{-1}	[6]
N/S-Ti_3C_2	2 mV s^{-1}	1 M Li_2SO_4	175 F g^{-1}	[7]
Ti_3C_2/TiO_2–nanoparticles	2 mV s^{-1}	6 M KOH	128 F g^{-1}	[8]
N-TiO_2/TiN/$Ti_3C_2T_x$-6	5 mV s^{-1}	1 M H_2SO_4	125 F g^{-1}	[9]
TiO_2/MXene/GO	10 A g^{-1}	1 M $LiPF_6$	78 mAh g^{-1}	[10]
HC-MXene/TiO_2	1 A g^{-1}	1 M $NaClO_4$	250 mAh g^{-1}	[11]
2D/2D TiO_2/MXene	1 A g^{-1}	1 M $LiPF_6$	55 mAh g^{-1}	[12]
N-TiO_{2-x}/C	0.1 A g^{-1}	1 M NaCl	23.6 mAh g^{-1}	[13]
TiO_2/Ti_3C_2	10 mV s^{-1}	1 M NaCl	207 F g^{-1}	This work
	1 mV s^{-1}	1 M KOH	93 F g^{-1}	
	1 mV s^{-1}	1 M Na_2SO_4	233 F g^{-1}	
	1 mV s^{-1}	1 M H_2SO_4	528 F g^{-1}	
	1 mV s^{-1}	1M Li_2SO_4	123 F g^{-1}	
	1 mV s^{-1}	1 M $LiPF_6$	173 F g^{-1}	

Table S3 Internal resistance (R_s) and charge transfer resistance (R_{ct}) of Ti_3C_2 and TiO_2/Ti_3C_2

Sample	R_s (Ω)	R_{ct} (Ω)
Ti_3C_2	1.33	1.25
TiO_2/Ti_3C_2	1.42	0.81

Table S4 Summary of salt desalination performance of MXene-based electrodes materials.

**MXene-based electrode**	**Operation mode**	**Initial concentr-ation (mg L^{-1})**	**Applied voltage/Current density**	**SAC (mg g^{-1})**	**SAR (mg g^{-1} min^{-1})**	**Cycle stability**	**Energy Consum-ption**	**Ref.**
**NaOH-$Ti_3C_2T_x$**	Flow-by CDI	100	1.2 V	12.19	-	20	-	[14]
**PPy-NiCo-LDH@MXene**	Flow-by CDI	200	1.2 V	31.5	4.7	40	-	[15]
**Ti_3C_2-MXene**	Flow-by CDI	292	1.2 V	13±2	1	30	-	[16]
**Fe_3O_4@Ti_3C_2**	Flow-by MCDI	500	1.2 V	44	1.47	40	-	[17]
**mPDA/MXene**	Flow-by MCDI	1000	1.5 V	37.72	1.27	200	0.69 kWh kg^{-1} NaCl	[18]
**poly(vinylalcohol)/$Ti_3C_2T_x$**	Flow-by MCDI	1000	10 mA g^{-1}	41.4	-	100	25 KWh kg^{-1} NaCl	[19]
**N–$Ti_3C_2T_x$**	Flow-by	5000	1.2 V	43.5±	-	24	-	[20]

	CDI			1.7				
**MXene/rGO**	Flow-by CDI	135	1.2 V	48	4.8	10	-	[21]
**Functional-ized MXene**	Flow-by CDI	5000	1.2 V	49	2.92	100	0.38 kWh kg^{-1} NaCl	[22]
**MXene/BC**	Flow-by CDI	585	1.2 V	12.27	1.23	20	63 $Wh\cdot m^{-3}$	[23]
**Cellulose fibers/ $Ti_3C_2T_x$ MXene**	Flow-by CDI	600	1.2 V	34	0.81	10	53.5 kT/ion	[24]
**$W_{18}O_{49}/Ti_3C_2$**	Flow-by MCDI	500	1.2 V	29.25	0.97	10	0.5642 kWh kg^{-1} NaCl	[25]
**MXene@COF**	Flow-by CDI	500	1.2 V	24.5	0.81	100	-	[26]
**MoS_2@MXene**	Flow-by CDI	500	1.2 V	35.6	2.6	40	0.544 kWh kg^{-1}	[27]

							NaCl	
**$Ti_3C_2T_x$ film**	Flow-by MCDI	585	20 mA·g^{-1}	67.7	0.78	50	0.24 kWh kg^{-1} NaCl	[28]
**TiO_2/Ti_3C_2**	Flow-by CDI	500	15 mA g^{-1}	55.3	0.63	--	--	This work
	Flow-by MCDI	500	15 mA g^{-1}	75.62	0.68	40	0.49 kWh kg^{-1} NaCl (12.42 Wh m^{-3})	
	Flow-by MCDI	500	1.2 V	64.32	1.07	--	--	

References

[1] J. Zhu, Y. Tang, C. Yang, F. Wang, M. Cao, *Journal of The Electrochemical Society* **2016**, 163, A785.

[2] Z. Li, Y. Liu, D. Guo, R. Jia, X. Zhai, L. Zhang, *Materials Research Express* **2019**, 6, 065056.

[3] W. XI, H. LI, *Journal of Inorganic Materials* **2021**, 36, 283.

[4] X. Lu, J. Zhu, W. Wu, B. Zhang, *Electrochimica Acta* **2017**, 228, 282.

[5] R. Liu, W. Guo, B. Sun, J. Pang, M. Pei, G. Zhou, *Electrochimica Acta* **2015**, 156, 274.

[6] W. Bao, L. Liu, C. Wang, S. Choi, D. Wang, G. Wang, *Advanced Energy Materials* **2018**, 8, 1702485.

[7] C. Yang, W. Que, Y. Tang, Y. Tian, X. Yin, *Journal of the Electrochemical Society* **2017**, 164, A1939.

[8] M. Cao, F. Wang, L. Wang, W. Wu, W. Lv, J. Zhu, *Journal of The Electrochemical Society* **2017**, 164, A3933.

[9] B. Yang, Y. She, C. Zhang, S. Kang, J. Zhou, W. Hu, *Nanomaterials* **2020**, 10, 345.

[10] R. Niu, R. Han, S. Tang, J. Zhu, *Journal of Power Sources* **2022**, 542, 231738.

[11] P. Gao, H. Shi, T. Ma, S. Liang, Y. Xia, Z. Xu, S. Wang, C. Min, L. Liu, *ACS Applied Materials & Interfaces* **2021**, 13, 51028.

[12] R. Niu, R. Han, Y. Wang, L. Zhang, Q. Qiao, L. Jiang, Y. Sun, S. Tang, J. Zhu, *Chemical Engineering Journal* **2021**, 405, 127049.

[13] M. Liang, X. Bai, F. Yu, J. Ma, *Nano Research* **2021**, 14, 684.

[14] B. Chen, A. Feng, R. Deng, K. Liu, Y. Yu, L. Song, *ACS Applied Materials & Interfaces* **2020**, 12, 13750.

[15] Y. Cai, Y. Wang, R. Fang, J. Wang, *Separation and Purification Technology* **2022**, 280, 119828.

[16] P. Srimuk, F. Kaasik, B. Krüner, A. Tolosa, S. Fleischmann, N. Jäckel, M. C. Tekeli, M. Aslan, M. E. Suss, V. Presser, *Journal of Materials Chemistry A* **2016**, 4, 18265.

[17] K. Wang, L. Chen, G. Zhu, X. Xu, L. Wan, T. Lu, L. Pan, *Desalination* **2022**, 522, 115420.

[18] Q. Li, X. Xu, J. Guo, J. P. Hill, H. Xu, L. Xiang, C. Li, Y. Yamauchi, Y. Mai, *Angewandte Chemie International Edition* **2021**, 60, 26528.

[19] J. Ai, J. Li, K. Li, F. Yu, J. Ma, *Chemical Engineering Journal* **2021**, 408, 127256.

[20] A. Amiri, Y. Chen, C. Bee Teng, M. Naraghi, *Energy Storage Materials* **2020**, 25, 731.

[21] H. Xu, M. Li, S. Gong, F. Zhao, Y. Zhao, C. Li, J. Qi, Z. Wang, H. Wang, X. Fan, W. Peng, J. Liu, *Journal of Colloid and Interface Science* **2022**, 624, 233.

[22] Z. Bo, Z. Huang, C. Xu, Y. Chen, E. Wu, J. Yan, K. Cen, H. Yang, K. Ostrikov, *Energy Storage Materials* **2022**, 50, 395.

[23] B. Li, K. Sun, W. Xu, X. Liu, A. Wang, S. Boles, B. Xu, H. Hu, D. Yao, *Nano Research* **2022**.

[24] S. Anwer, D. H. Anjum, S. Luo, Y. Abbas, B. Li, S. Iqbal, K. Liao, *Chemical Engineering Journal* **2021**, 406, 126827.

[25] J. Liang, J. Yu, W. Xing, W. Tang, N. Tang, J. Guo, *Chemical Engineering Journal* **2022**, 435, 134922.

[26] S. Zhang, X. Xu, X. Liu, Q. Yang, N. Shang, X. Zhao, X. Zang, C. Wang, Z. Wang, J. G. Shapter, Y. Yamauchi, *Materials Horizons* **2022**, 9, 1708.

[27] Y. Cai, Y. Wang, L. Zhang, R. Fang, J. Wang, *ACS Applied Materials & Interfaces* **2022**, 14, 2833.

[28] J. Ma, Y. Cheng, L. Wang, X. Dai, F. Yu, *Chemical Engineering Journal* **2020**, 384, 123329.
